# The Influencing Contexts and Potential Mechanisms Behind the Use of Web-Based Self-management Support Interventions: Realistic Evaluation

**DOI:** 10.2196/34925

**Published:** 2022-07-01

**Authors:** Marscha Engelen, Betsie van Gaal, Hester Vermeulen, Rixt Zuidema, Sebastian Bredie, Sandra van Dulmen

**Affiliations:** 1 IQ Healthcare Radboud Institute for Health Sciences Radboud university medical center Nijmegen Netherlands; 2 School of Health Studies HAN University of Applied Sciences Nijmegen Netherlands; 3 Research Group Proactive Care for Older People Faculty of Health Care University of Applied Sciences Utrecht Utrecht Netherlands; 4 Division of Vascular Medicine Department of Internal Medicine Radboud university medical center Nijmegen Netherlands; 5 Department of Primary and Community Care Radboud Institute for Health Sciences Radboud university medical center Nijmegen Netherlands; 6 Nivel - Netherlands institute for health services research Department of Communication in healthcare Utrecht Netherlands; 7 Faculty of Caring Science Work Life and Social Welfare University of Borås Borås Sweden

**Keywords:** self-management, telemedicine, chronic disease, cardiovascular diseases, rheumatoid arthritis, patient dropouts, realistic evaluation, program use

## Abstract

**Background:**

Self-management can increase self-efficacy and quality of life and improve disease outcomes. Effective self-management may also help reduce the pressure on health care systems. However, patients need support in dealing with their disease and in developing skills to manage the consequences and changes associated with their condition. Web-based self-management support programs have helped patients with cardiovascular disease (CVD) and rheumatoid arthritis (RA), but program use has been low.

**Objective:**

This study aimed to identify the patient, disease, and program characteristics that determine whether patients use web-based self-management support programs or not.

**Methods:**

A realistic evaluation methodology was used to provide a comprehensive overview of context (patient and disease characteristics), mechanism (program characteristics), and outcome (program use). Secondary data of adult patients with CVD (n=101) and those with RA (n=77) were included in the study. The relationship between context (sex, age, education, employment status, living situation, self-management [measured using Patient Activation Measure-13], quality of life [measured using RAND 36-item health survey], interaction efficacy [measured using the 5-item perceived efficacy in patient-physician interactions], diagnosis, physical comorbidity, and time since diagnosis) and outcome (program use) was analyzed using logistic regression analyses. The relationship between mechanism (program design, implementation strategies, and behavior change techniques [BCTs]) and outcome was analyzed through a qualitative interview study.

**Results:**

This study included 68 nonusers and 111 users of web-based self-management support programs, of which 56.4% (101/179) were diagnosed with CVD and 43.6% (78/179) with RA. Younger age and a lower level of education were associated with program use. An interaction effect was found between program use and diagnosis and 4 quality of life subscales (social functioning, physical role limitations, vitality, and bodily pain). Patients with CVD with higher self-management and quality of life scores were less likely to use the program, whereas patients with RA with higher self-management and quality of life scores were more likely to use the program. Interviews with 10 nonusers, 10 low users, and 18 high users were analyzed to provide insight into the relationship between mechanisms and outcome. Program use was encouraged by an easy-to-use, clear, and transparent design and by recommendations from professionals and email reminders. A total of 5 BCTs were identified as potential mechanisms to promote program use: tailored information, self-reporting behavior, delayed feedback, providing information on peer behavior, and modeling.

**Conclusions:**

This realistic evaluation showed that certain patient, disease, and program characteristics (age, education, diagnosis, program design, type of reminder, and BCTs) are associated with the use of web-based self-management support programs. These results represent the first step in improving the tailoring of web-based self-management support programs. Future research on the interaction between patient and program characteristics should be conducted to improve the tailoring of participants to program components.

## Introduction

Chronic diseases are a major burden for patients, and the growing number of people with (several) chronic conditions puts a strain on our health care systems. The pressure on health care services may be decreased and the quality of life of people with chronic conditions may be improved if these individuals can self-manage their condition and adapt to their situation [1-3]. Self-management is defined as “the individual’s ability to manage the symptoms, treatment, physical consequences, psychological consequences and lifestyle changes inherent in living with a chronic condition” [4]. This is not easy for patients with chronic conditions because they may not feel confident enough to manage their disease [5,6]. Factors such as disease burden, comorbidities, and competing life circumstances can impair a patient’s capacity to self-manage their condition. These obstacles can be overcome with the help of health care professionals, support staff, peers, or digital support programs.

Self-management support interventions have already been developed for a broad range of long-term medical conditions and have shown improvements in self-management and other health outcomes [7,8]. However, it is challenging to establish self-management support that is feasible for both patients and health care professionals. Web-based self-management support programs may overcome these barriers by providing disease-specific information and personal feedback and by monitoring behavior [9]. Web-based interventions have become more frequent in response to the COVID-19 pandemic, and health care professionals and patients have become more open to digital solutions. Adherence to and uptake of web-based interventions are essential for increasing self-management. However, despite advantages such as easy accessibility and anonymity, studies have shown that the use of and exposure to web-based self-management interventions are unsatisfactory [10,11].

We recently developed 2 comprehensive, multicomponent, and theory-based web-based self-management interventions using the intervention mapping framework [12]: one for patients with cardiovascular disease (CVD) called Vascular View and one for patients with rheumatoid arthritis (RA) called Coping with RA. Both programs were developed in close collaboration with patients and health care professionals to promote their use and meet patients’ needs. These programs have been described in detail elsewhere [13-16]. Unexpectedly, explorative randomized controlled trials showed no effect of these programs on self-management, possibly because patients were not using them, even though we tried to match them to patients’ needs. Vascular View was used by 62.4% (65/101) of the intervention group and Coping with RA by 63% (50/78) of the intervention group. This phenomenon of participants dropping out of or not using an intervention is called *the law of attrition* and is a major challenge when developing and evaluating eHealth interventions [17].

There are many reasons why participants use or do not use a web-based self-management program. Patient characteristics, such as older age, lower education levels, and lower income, have been associated with lower eHealth use [18-20]. Self-management ability, self-efficacy, and quality of life may also be influencing factors, as they are associated with self-management [21,22]. The use of eHealth interventions demands that patients take control of their chronic diseases; therefore, a basic level of self-management is a prerequisite for the use of web-based interventions. Disease characteristics, such as disease burden, may also influence program use. For example, although some patients with CVD do not experience physical symptoms, they still have to adapt their daily routine by making lifestyle changes and taking medication. Patients with CVD might also experience psychosocial consequences, such as being anxious about a secondary cardiovascular event. RA has a more direct physical impact on patients with symptoms such as pain, stiff joints, and fatigue. RA also has psychosocial consequences on patients, such as changes in social roles and feelings of depression. These differences between CVD and RA may influence the self-management needs and program use of these patients. Finally, program characteristics, such as the type of information or applied implementation strategies, may influence whether a patient uses the program [23,24].

In this study, we identified the patient, disease, and program characteristics that determine whether patients with CVD and patients with RA use the Vascular View and Coping with RA web-based self-management support programs. The findings can be used to tailor web-based self-management support programs to individual patients and thereby increase their use.

## Methods

### Design

**Figure 1:**
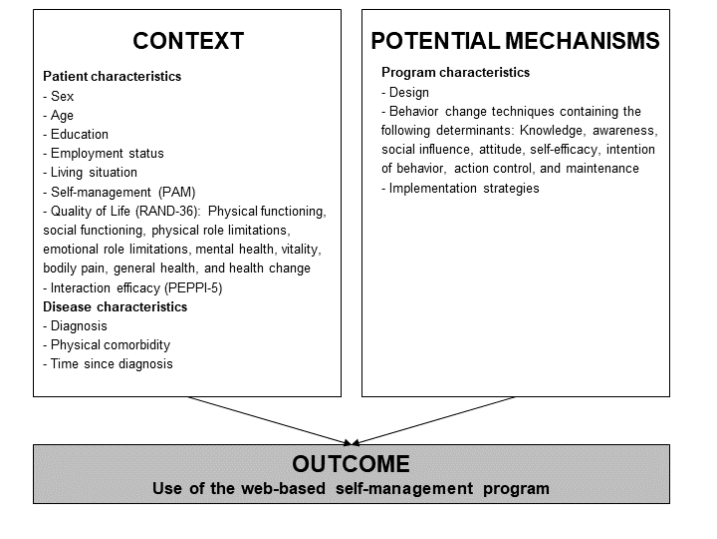
Realistic evaluation: context, potential mechanisms, and outcome. PAM: Patient Activation Measure; PEPPI-5: 5-item version perceived efficacy in patient-physician interactions; RAND-36: RAND 36-item health survey.

The realist evaluation methodology was used to gain a comprehensive understanding of why patients use or do not use web-based self-management support interventions [25]. We structured the analysis using the context-mechanism-outcome configuration to identify contextual factors (features of the conditions, eg, patient characteristics, that influence the intervention mechanisms) and potential mechanisms (what and how intervention components are responsible for change) that affect the intervention outcome (Figure 1). The identified mechanisms are described as “potential” as it was beyond the scope of this study to also test their effectiveness. In this study, we used data from 2 previous studies [14,16] that were approved by the medical ethics committee of Arnhem-Nijmegen in the Netherlands (No. 2015-1908 for CVD and 2014-1208 for RA).

### Context: Patient and Disease Characteristics

#### Overview

This study included data of 2 patient groups with a chronic disease. Patients in the CVD group had experienced a myocardial infarction, cerebrovascular disease, or peripheral artery disease or a combination of these within 2 months to 1 year of the study starting. Patients in the RA group were diagnosed with RA, a chronic autoimmune disease that predominantly affects the joints, before the start of the study. The baseline data were collected at the start of each study. Inclusion criteria were as follows: (1) aged ≥18 years; (2) ability to read and understand Dutch; (3) access to a computer, internet, and email account; and (4) not receiving psychiatric or psychological treatment.

#### Measurements

The included patient and disease characteristics are expected to be associated with self-management and might, therefore, be related to program use. The following patient characteristics were studied to determine whether they were associated with program use: sex (male or female), age (years), education (low: no education, primary education, or lower secondary education; intermediate: secondary vocational education; and high: higher education or university), work participation (yes or no), living situation (alone or together), self-management, quality of life, and communication efficacy. Self-management was measured using the Patient Activation Measure (PAM-13), which includes statements about an individual’s knowledge, confidence, and skills for self-management of their behavior in response to their chronic illness and about their level of activation. The PAM-13 scores 13 items on a 5-point scale, with a higher score indicating a higher level of patient activation [26,27]. Quality of life was measured using the RAND 36-item health survey (RAND-36), which contains 36 items measuring 8 dimensions: physical functioning, social functioning, physical role limitations, emotional role limitations, mental health, vitality, pain, and general health perception [28]. A higher score indicates a better quality of life. Communication efficacy was measured using the 5-item perceived efficacy in patient-physician interactions, which scores 5 items on a 5-point Likert scale that are summed to determine the total score. A higher score reflects greater confidence in interactions with the health care professional [29,30]. A total of 3 disease characteristics were included: diagnosis (CVD or RA), time since diagnosis (years), and physical comorbidity (yes or no).

#### Mechanism: Program Characteristics

A total of 2 comprehensive, multicomponent, web-based self-management programs were studied for this realistic evaluation: Vascular View and Coping with RA. Multimedia Appendix 1 describes the characteristics of both the programs. There were similarities between the 2 programs and their execution. First, both the programs used the same web-based platform and program design. Second, health care professionals working in one hospital were asked to invite patients to participate in the study. Third, participants had unlimited access to the programs for 12 months between December 2014 and October 2016 and could use the program modules in any sequence and as often as they wanted. A total of 3 program characteristics were considered as potential mechanisms in this realistic evaluation: design, behavior change techniques (BCTs), and implementation strategies [31].

Vascular View was developed for patients with CVD [13] and contained six modules: (1) coping with CVD and its consequences, (2) setting boundaries in daily life, (3) lifestyle, (4) healthy nutrition, (5) being physically active in a healthy way, and (6) interaction with health professionals. Relevant BCTs (Table 1) were translated to practical applications including general written information on the disease, reading quotes and watching videos of other patients with CVD as role models and receiving personalized feedback, and encouraging participants to write in diaries and perform exercises. Patients filled out a questionnaire to read which modules were recommended for them and received feedback after filling out a lifestyle questionnaire. The implementation strategies were applied in 4 ways. First, the patients received a written instruction manual and digital promotion flyer at the start of the program. Second, they received 1 telephone reminder if they had not used the program within 3 months. Third, they received email reminders if they had started modules but left them incomplete. Finally, a newsletter was sent every 2 months to all participants to informally remind them of the program.

Coping with RA was developed for patients with RA [15]. The program contained the following nine modules that dealt with health-related problems: (1) balancing rest and activity, (2) setting boundaries, (3) asking for help and support, (4) using medicines, (5) communicating with health professionals, (6) using assistive devices, (7) performing physical exercises, (8) coping with worries, and (9) coping with RA. BCTs (such as providing general information on the disease, self-monitoring, persuasive communication, modeling, self-persuasion, and tailoring) were translated into practical applications (such as texts, videos, exercises, and a medication intake schedule). The content of each program module was tailored to the specific user based on a questionnaire filled out at the start of the web-based program. A total of 3 implementation strategies were applied. First, health care professionals were asked to inform their patients about the web-based program during the consultation. Second, patients received a written instruction manual at the start of the program. Third, patients received biweekly email reminders to use the program.

**Table 1:** Overview of applied determinants and behavior change techniques per program.

Determinant	Behavior change techniques	Vascular View	Coping with RA^a^
Knowledge	Provide general information about health behavior	✓^b^	✓
Knowledge	Increase memory and/or understanding of transferred information	✓	✓
Awareness	Risk communication	✓	✓
Awareness	Self-monitoring of behavior	✓	✓
Awareness	Self-report of behavior	N/A^c^	✓
Awareness	Delayed feedback of behavior	✓	N/A
Social influence	Provide information about peer behavior	✓	✓
Social influence	Mobilize social norm	✓	N/A
Attitude	Re-evaluation of outcomes and self-evaluation	✓	N/A
Attitude	Persuasive communication	✓	✓
Attitude	Reward behavioral progress	N/A	✓
Self-efficacy	Modeling	✓	✓
Self-efficacy	Practice and guided practice	✓	✓
Self-efficacy	Plan coping response	N/A	✓
Self-efficacy	Graded tasks and goal setting	✓	N/A
Self-efficacy	Reattribution training and external attribution of failure	✓	N/A
Intention of behavior	General intention formation	✓	N/A
Intention of behavior	Develop medication schedule	N/A	✓
Intention of behavior	Specific goal setting	✓	N/A
Intention of behavior	Review of general and/or specific goals	✓	N/A
Intention of behavior	Use of social support	N/A	✓
Action control	Use of cues	N/A	✓
Action control	Self-persuasion	N/A	✓
Maintenance	Goals for maintenance	✓	N/A

^a^RA: rheumatoid arthritis.

^b^✓: The behavior change technique was included in the program.

^c^N/A: not applicable.

### Outcome: Program Use

Program use was a dichotomous outcome and was divided into nonusers (0 or 1 visit) and users (≥2 visits). The cut-off point between users and nonusers was arbitrarily set at 2 visits because this was seen as a reflection of whether a patient would have had the opportunity to benefit from the program.

### Analysis

#### Relation Between Context and Outcome: Quantitative Analysis

All quantitative data were analyzed using SPSS Statistics (version 25; IBM Corp). Descriptive analyses were used to describe the patient and disease characteristics of nonusers and users. Differences between the characteristics were tested using 2-tailed *t* tests and chi-square tests. A 2-sided *P* value of <.05 was considered statistically significant in all analyses.

Logistic regression analyses were used to determine which characteristics were associated with program use. Program use (nonuser or user) was the dependent factor. Patient and disease characteristics (sex, age, education, employment status, living situation, self-management, quality of life, interaction efficacy, diagnosis, physical comorbidity, and time since diagnosis) were tested as possible factors. The strength of the relations was interpreted using odds ratios with 95% CIs. Factors with a *P* value of <.20 were tested in the final model. The model adequacy in the bivariate logistic regression was confirmed with a backward likelihood ratio test. As this is an explorative analysis, the Bonferroni correction was not applied to counteract the problem of multiple variables.

Sensitivity analysis was performed to compare the characteristics of users and nonusers in the CVD and RA groups. Logistic regression analyses were performed for all characteristics (sex, age, education, employment status, living situation, self-management, quality of life, interaction efficacy, diagnosis, physical comorbidity, and time since diagnosis) and for diagnosis, characteristic, and diagnosis×characteristic. These analyses determined whether there was an interaction between the diagnosis and characteristic. The strengths of the relationships were interpreted using odds ratios with 95% CI.

#### Relation Between Mechanism and Outcome: Qualitative Analysis

As a sequence of efficacy studies of Vascular View and Coping with RA, interviews were conducted to provide insight into (1) why patients used or did not use the web-based program and (2) the experiences with the web-based program among users. The results of the qualitative study on the Coping with RA program have been described elsewhere [23]. In this study, we focused on a part of the interviews to determine potential program characteristics.

A random selection of Vascular View and Coping with RA users and nonusers were invited for an interview after data on the explorative randomized controlled trials were collected. Purposive sampling was used to select patients regarding the degree to which they used the program. The participants were divided into 3 groups: nonusers, low users, and high users. After providing written consent, each patient was interviewed once via telephone. Semistructured interviews, lasting no longer than 30 minutes, were audio-recorded and anonymized. Interviews were transcribed verbatim and transferred to Excel (Microsoft). A total of 3 themes were determined beforehand: Program design, implementation strategies, and BCTs. The first researcher (ME) thematically analyzed the interviews to identify the potential program characteristics that influence use. First, the verbatim text was read and the relevant parts were marked. Next, the researcher determined barriers and facilitating factors for program use, which were divided into the 3 themes.

## Results

### Overview

**Figure 2:**
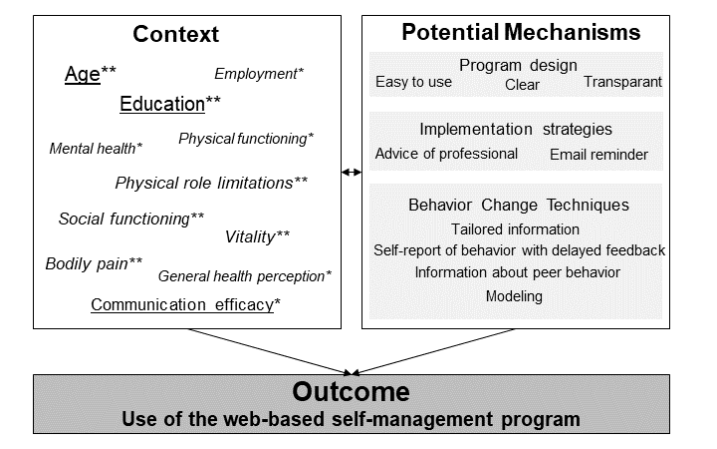
Overview of patient and disease characteristics (context) and program characteristics (potential mechanisms) that influence program use (outcome). Underlined variables are factors associated with program use; italicized variables are factors associated with program use in the interaction effect with diagnosis; and the font size reflects the degree of prediction; *P<.20; **P<.05.

We investigated the relations between context, mechanism, and outcome to determine which factors are associated with the use of a web-based self-management support program. Figure 2 summarizes the patient, disease, and program characteristics that influence program use.

### Relation Between Context (Patient and Disease Characteristics) and Outcome (Program Use)

#### Descriptive Data

To analyze the relation between patient and disease characteristics (context) and program use (outcome), 68 patients were defined as nonusers and 111 were defined as users. More users were diagnosed with CVD (63/111, 56.8%) than with RA (48/111, 43.2%). Patient and disease characteristics of the nonuser and user groups are presented in Table 2.

**Table 2:** Characteristics of the users and nonusers in the total group, cardiovascular disease (CVD) group, and rheumatoid arthritis (RA) group.

Characteristics		Total group	CVD group	RA group
**Sex (user), n (%)**				
	Male	59 (59.6)	42 (58.3)	17 (63.0)
	Female	52 (65)	21 (72.4)	31 (60.8)
**Level of education (user), n (%)**				
	Low	21 (77.8)	14 (82.4)	7 (70.0)
	Intermediate	40 (51.9)	16 (47.1)	24 (55.8)
	High	50 (66.7)	33 (66.0)	17 (68)
**Work participation (user), n (%)**				
	Yes	51 (66.2)	24 (60.0)	27 (73.0)
	No	60 (58.8)	39 (63.9)	21 (51.2)
**Living situation (user), n (%)**				
	Alone	19 (61.3)	10 (62.5)	9 (60.0)
	Together	92 (62.2)	53 (62.4)	39 (61.9)
**Physical comorbidity (user), n (%)**				
	Yes	50 (61.0)	24 (60.0)	26 (61.9)
	No	61 (62.9)	39 (63.9)	22 (61.1)
**Age (years), mean (SD)**				
	Nonusers	64.5 (10.0)^a^	65.1 (9.7)^b^	63.8 (10.5)^c^
	Users	60.5 (10.4)^d^	61.5 (9.4)^e^	59.2 (11.6)^f^
**Time since diagnosis (years), mean (SD)**				
	Nonusers	8.7 (10.6)^g^	5.0 (7.9)^h^	13.4 (11.9)^c^
	Users	8.1 (10.6)^d^	3.8 (7.7)^e^	13.8 (11.2)^f^
**Self-management score, PAM-13^i^, mean (SD)**				
	Nonusers	40.2 (4.8)^j^	40.7 (4.4)^b^	39.5 (5.4)^k^
	Users	40.4 (5.5)^l^	40.4 (5.5)^e^	40.3 (5.6)^m^
**Interaction efficacy, PEPPI-5^n^, mean (SD)**				
	Nonusers	21.4 (3.3)^j^	21.3 (2.8)^b^	21.5 (3.8)^k^
	Users	20.5 (3.3)^d^	20.0 (3.6)^e^	21.1 (2.9)^f^
**Physical functioning, RAND-36^o^, mean (SD)**				
	Nonusers	64.0 (27.8)^a^	71.3 (25.3)^b^	54.3 (28.3)^c^
	Users	68.8 (25.1)^d^	70.9 (26.0)^p^	66.1 (23.9)^f^
**Social functioning, RAND-36, mean (SD)**				
	Nonusers	71.9 (24.1)^a^	77.6 (22.2)^b^	64.6 (24.8)^k^
	Users	74.9 (22.4)^d^	74.4 (26.0)^p^	75.5 (16.7)^f^
**Role physical, RAND-36, mean (SD)**				
	Nonusers	51.1 (43.9)^a^	62.5 (41.0)^b^	36.7 (43.9)^k^
	Users	56.8 (41.1)^d^	56.7 (41.2)^e^	56.8 (41.5)^f^
**Role emotional, RAND-36, mean (SD)**				
	Nonusers	75.1 (40.3)^a^	75.4 (40.0)^b^	74.7 (41.5)^c^
	Users	80.8 (34.1)^d^	78.8 (35.1)^e^	83.3 (33.0)^f^
**Mental health, RAND-36, mean (SD)**				
	Nonusers	75.5 (17.0)^a^	78.1 (17.4)^b^	72.1 (16.1)^k^
	Users	76.4 (13.9)^d^	75.7 (15.4)^e^	77.4 (11.7)^f^
**Vitality, RAND-36, mean (SD)**				
	Nonusers	58.4 (21.2)^a^	62.5 (19.2)^b^	53.1 (22.9)^k^
	Users	57.5 (18.9)^d^	56.1 (20.4)^e^	59.3 (16.9)^f^
**Bodily pain, RAND-36, mean (SD)**				
	Nonusers	70.0 (26.9)^a^	80.2 (23.5)^b^	56.9 (25.5)^k^
	Users	72.6 (21.8)^d^	75.4 (23.5)^e^	68.9 (19.0)^f^
**General health, RAND-36, mean (SD)**				
	Nonusers	51.3 (19.1)^a^	55.4 (18.1)^b^	46.0 (19.4)^c^
	Users	53.5 (19.0)^d^	53.3 (19.9)^e^	53.8 (17.8)^f^
**Health change, RAND-36, mean (SD)**				
	Nonusers	47.8 (22.3)^a^	51.3 (23.2)^b^	43.3 (20.7)^k^
	Users	51.6 (24.8)^d^	52.4 (25.3)^e^	50.5 (24.5)^f^

^a^n=67.

^b^n=38.

^c^n=29.

^d^n=111.

^e^n=63.

^f^n=48.

^g^n=66.

^h^n=37.

^i^PAM-13: Patient Activation Measure.

^j^n=68.

^k^n=30.

^l^n=110.

^m^n=47.

^n^PEPPI-5: 5-item perceived efficacy in patient-physician interactions.

^o^RAND-36: RAND 36-item health survey.

^p^n=63.

#### Main Analysis of the Relation Between Context and Outcome

Univariate analyses showed that age, education, and communication efficacy with health care professionals (5-item perceived efficacy in patient-physician interactions) were associated with the use of web-based self-management interventions (Table 3). Table 2 shows that younger patients (mean 60.5, SD 10.4 years) and patients with a lower level of education (21/27, 78% used the intervention) were more likely to use the program than older patients (mean 64.5, SD 10.0 years) and patients with an intermediate level of education (40/77, 52% used the intervention). Furthermore, users scored lower on communication efficacy with health care professionals (mean 20.5, SD 3.3) than nonusers (mean 24.4, SD 3.3). A combination of age and education level provided the best model for predicting the use of the web-based self-management program (Table 4) and correctly predicted whether a person would be a user or nonuser in 69.1% (123/179) of cases. Users were correctly predicted in 91.9% (102/111) of cases and nonusers in 31% (21/68) of cases.

**Table 3:** Results of the univariate logistic regressions for all possible factors for total group.

	OR^a^ (95% CI)	*P* value
Sex	0.79 (0.43-1.46)	.46
Age	0.96 (0.93-0.99)	.02^b^
Education (reference: low)—intermediate	0.31 (0.11-0.85)	.02^b^
Education (reference: low)—high	0.57 (0.21-1.60)	.29
Employment status	1.37 (0.74-2.54)	.31
Living situation	1.04 (0.47-2.30)	.93
Diagnosis	0.97 (0.53-1.77)	.97
Physical comorbidity	0.92 (0.50-1.69)	.79
Time since diagnosis	1.00 (0.97-1.02)	.72
Self-management (PAM^c^)	1.01 (0.95-1.07)	.77
Communication efficacy (PEPPI^d^)	0.92 (0.83-1.01)	.08^e^
Physical functioning (RAND-36^f^)	1.01 (1.00-1.02)	.23
Social functioning (RAND-36)	1.01 (0.99-1.02)	.40
Role physical (RAND-36)	1.00 (1.00-1.01)	.38
Role emotional (RAND-36)	1.00 (1.00-1.01)	.32
Mental health (RAND-36)	1.00 (0.98-1.02)	.68
Vitality (RAND-36)	1.00 (0.98-1.01)	.78
Bodily pain (RAND-36)	1.01 (0.99-1.02)	.47
General health (RAND-36)	1.01 (0.99-1.02)	.47
Health change (RAND-36)	1.01 (0.99-1.02)	.30

^a^OR: odds ratio.

^b^*P*<.05.

^c^PAM: Patient Activation Measure.

^d^PEPPI-5: 5-item perceived efficacy in patient-physician interactions.

^e^*P*<.20.

^f^RAND-36: RAND 36-item health survey.

**Table 4:** Final model of factors associated with the use of web-based self-management programs^a^.

	B	SE	OR^b^ (95% CI)	*P* value
Constant	3.58	1.16	N/A^c^	.002
Age	−0.04	0.017	0.96 (0.93-1.00)	.03
Education (intermediate vs low)	−1.06	0.52	0.35 (0.12-0.96)	.04

^a^Nagelkerke R^2^=0.049.

^b^OR: odds ratio.

^c^N/A: not applicable.

#### Sensitivity Analysis of the Relation Between Context and Outcome

Sensitivity analysis showed a significant interaction between diagnosis and the RAND-36 subscales social functioning, physical role limitations, vitality, and bodily pain (Table 5). The descriptive data presented in Table 2 show that scores on self-management (PAM-13) and some quality-of-life subscales (RAND-36) were different between the CVD and RA groups. Patients with CVD with higher scores on self-management and quality of life were less likely to use the program. In contrast, patients with RA with higher scores on self-management and quality of life were more likely to use the program.

**Table 5:** Results of the interaction effects between diagnosis (cardiovascular disease and rheumatoid arthritis) and possible factors.

	OR^a^ (95% CI)	*P* value
Sex	2.06 (0.54-7.89)	.29
Age	1.00 (0.94-1.07)	.95
Education (reference: low)—intermediate	2.84 (0.37-22.06)	.32
Education (reference: low)—high	2.19 (0.27-17.98)	.47
Employment status	3.04 (0.87-10.66)	.08^b^
Living situation	1.09 (0.22-5.37)	.92
Physical comorbidity	1.22 (0.36-4.18)	.75
Time since diagnosis	1.02 (0.96-1.09)	.50
Self-management (PAM^c^)	1.04 (0.93-1.17)	.52
Communication efficacy (PEPPI^d^)	1.09 (0.90-1.33)	.39
Physical functioning (RAND-36^e^)	1.02 (0.99-1.04)	.14^b^
Social functioning (RAND-36)	1.03 (1.00-1.06)	.03^f^
Role physical (RAND-36)	1.02 (1.00-1.03)	.05^f^
Role emotional (RAND-36)	1.00 (0.99-1.02)	.64
Mental health (RAND-36)	1.04 (1.00-1.08)	.08^b^
Vitality (RAND-36)	1.03 (1.00-1.07)	.04^f^
Bodily pain (RAND-36)	1.04 (1.01-1.06)	.02^f^
General health (RAND-36)	1.03 (1.00-1.07)	.09^b^
Health change (RAND-36)	1.01 (0.99-1.04)	.37

^a^OR: odds ratio.

^b^*P*<.20.

^c^PAM: Patient Activation Measure.

^d^PEPPI: Perceived Efficacy in Patient-Physician Interactions.

^e^RAND-36: RAND 36-item health survey.

^f^*P*<.05.

### Relation Between Mechanisms (Program Design, Implementation Strategies, and BCTs) and Outcome (Program Use)

A random sample of study participants was interviewed to gain insight into why they did or did not use the web-based self-management program. In the CVD group, 6 nonusers, 6 low users, and 6 high users were interviewed. In the RA group, 4 nonusers, 4 low users, and 13 high users were interviewed. The results were divided into 3 themes: program design, implementation strategies, and BCTs. Table 6 provides quotes that show the barriers and facilitators for program use on the 3 themes: program design, implementation strategies, and BCTs.

Most interviewees were pleased with the program design. However, some experienced difficulties in using the program, and so they did not use it as often. A search function would make it easier to find relevant information. Several users and nonusers stated that they had overlooked parts of the program; for example, 1 participant only used the diaries because he did not know that training modules were available. Another major reason for not using the program were problems with logging in. These observations indicate that ease of use was an important factor for program use among our respondents.

Explanations given by the respondents as to why they did or did not use the program also revealed factors affecting program use. Several respondents stated that they did not participate for their own benefit but rather to facilitate scientific research. Others used the program following advice from their health care professional or because they were curious and wanted to better understand their disease. The biweekly reminders to fill out the diaries in the Coping with RA program helped many respondents to use the diaries.

**Table 6:** Quotes from the interviews with users and nonusers.

	Barriers	Facilitators
Program design	“Well, I couldn’t log in. Somehow I really couldn’t, or it wasn’t clear to me. Through the internet I find it very difficult to do.” (Coping with RA^a^, participant 5)	“Yes I liked the lay-out. The information was orderly, you could easily click on what you wanted to see. So the program was very well organized.” (Coping with RA, participant 21)
Implementation strategies	Barriers for implementation were not described.	“The hospital nurse advised me to use the program.” (Vascular View, participant 1) “If I received an email that said I still had something to do, I always did.” (Coping with RA, participant 8)
Behavior change techniques	“The program only gives input but I missed feedback options, for example to keep track of my weight.” (Vascular View, participant 16)	“I wanted information on how to deal with my recent diagnosis.” (Vascular View, participant 7)

^a^RA: rheumatoid arthritis.

Comments related to program content were assigned to the relevant BCTs, and some of these BCTs were identified as potential mechanisms affecting program use. The first BCT (providing general information about health behavior) was often mentioned in the interviews. For example, respondents with a long disease history stated that the information was too general. Furthermore, some respondents saw on the overview page that none of the modules contained new or interesting information, and so they did not use the program further. Respondents reported that reliable information was a reason for using the program. The Vascular View program includes a physical activity and nutrition diary (for the self-monitoring of behavior BCT), which was rarely used. One respondent said they had missed a feedback function in the diaries and had already used other, more advanced, mobile apps instead. The pain and fatigue diaries in the Coping with RA program were used more often by respondents (for the self-report of behavior BCT). Patients appreciated the possibility of keeping track of their pain and fatigue and of receiving a graphical overview of their input (the delayed feedback of behavior BCT). Program users also liked the stories and videos of peers (which provided information about peer behavior BCT and modeling BCT). One respondent said that these made her feel recognized and supported and showed her that she was on the right track.

## Discussion

### Principal Findings

In this realistic evaluation of 2 web-based self-management interventions, we searched for patient, disease, and program characteristics that determine whether patients will use the programs. Regarding the relationship between context and outcome, patient and disease characteristics, younger age, and lower level of education were associated with program use. In addition, 4 quality of life subscales (social function, physical role limitations, vitality, and bodily pain) interacted significantly with the diagnosis group to affect program use. Regarding the relationship between Potential Mechanisms (program characteristics) and outcome, participants indicated that an easy-to-use, clear, and transparent design would motivate them to use the program. Email reminders and recommendations from health care professionals were found to be potential implementation mechanisms for promoting program use. The top five BCT techniques that encouraged interviewees to use the program were (1) tailored information, (2) self-report of behavior, (3) delayed feedback, (4) information about peer behavior, and (5) modeling.

### Tailoring Web-Based Self-management Interventions to Increase Program Use

Our findings show that patient and disease characteristics can be used to tailor web-based self-management interventions and, therefore, increase their use. Younger age increased program use in our study, which is in agreement with the results of previous studies. However, in contrast to our finding that a lower level of education increased program use, earlier studies showed that a higher level of education increased program use [18-20,32,33]. Despite this discrepancy, these results show that age and education both influence program use, possibly because they are both related to eHealth literacy. eHealth literacy is the ability to seek, find, understand, and appraise health information from electronic sources and to use this knowledge to address or solve a health problem [34]. Concerns have been raised about a *digital divide*, which is the gap between patients who are able to use eHealth and those who are not [19]. Our study emphasizes the need to pay attention to these issues, as both age and education are strongly related to eHealth literacy [35], and eHealth literacy is needed to benefit from web-based interventions. Different forms of self-management support should be provided to people with low eHealth literacy.

Disease burden can be both mental and physical and is another possible factor related to the use of web-based self-management support programs. Patients with RA have a lower physical quality of life and experience more pain than those with CVD. Individuals with episodic or deteriorating diseases such as RA have different self-management support needs than those with stable chronic diseases [36,37]. Patients with CVD have reported fewer self-management support needs than those with other chronic diseases because their disease as a smaller impact on their live [38]. These variations in the perceived burden of disease can affect the motivation to change. A higher perceived disease burden has been associated with a higher perception of the necessity for treatment, which increases adherence to treatment [39]. We have shown this in this study; in the RA group, users rated their physical quality of life as higher than nonusers, whereas in the CVD group, users rated their physical quality of life as lower than nonusers. This suggests that a certain level of burden is needed to feel urgency and to be motivated to use a web-based self-management support program. However, a web-based intervention might not be sufficient when the disease burden is too high. In such cases, face-to-face support from health care professionals is recommended [23].

### The Influence of Program Characteristics

The study participants provided some recommendations for an effective web-based self-management support program. These recommendations included being easy to use, providing appropriate reminders, tailoring information to the user, allowing patients to self-report their behavior and receive delayed feedback, and providing information about peer behavior and modeling. These results are in line with those of a Delphi study that identified new information and the possibility of monitoring personal progress as important factors promoting the use of an eHealth self-management intervention [40]. In addition, previous research has shown that peer support and email or phone contact increase the use of eHealth interventions [10]. These observations suggest that adding an interactive component to our Vascular View and Coping with RA programs, which allows users to communicate with peers and health care professionals, may promote program use. Counselor support has been found to be important for program use in previous studies [10], and our participants stated that interaction would have stimulated them to use the program. The role of health care professionals should never be underestimated, especially as blended care (a combination of eHealth interventions and face-to-face consultations with a health care professional) increases the use of eHealth interventions, including more program components [41].

### One Size Does Not Fit All

Our results emphasize that one program will not be suitable for every patient. Self-management programs should be tailored to patients’ individual needs. It should also be noted that not all patients can use and benefit from web-based interventions. The validated Self-Management Screening (SeMaS) questionnaire can help identify potential barriers to self-management and can help health care professionals determine their patients’ support needs [42]. The factors affecting program use identified in this study were in accordance with the components of the SeMaS, including age, education, disease burden (both low and high disease burden can be barriers to self-management), computer skills, and social support. The SeMaS can help health care professionals to choose appropriate interventions and to decide which patients would benefit from a web-based self-management support intervention [43].

### The Use of a Realistic Evaluation

Given the complexity of web-based self-management interventions, realistic evaluations can reveal what makes an intervention work, which a simple cause-and-effect relationship between an intervention and its outcome may not be able to do. This is especially important for eHealth interventions because dropout and nonuse rates are high [17]. The aim of a realistic evaluation is to determine what works for whom, in what circumstances, and why. We tried to answer these questions by analyzing what patient and disease characteristics influence program use (context) and by describing what program characteristics influence program use (potential mechanisms; Figure 2). Unfortunately, we were not able to analyze the interaction between context and mechanism and how this affects the outcome. This should be addressed in future research to further improve the tailoring and effectiveness of eHealth interventions.

### Limitations

The findings of our realistic evaluation should be considered in the context of several limitations. The principal limitation was that we used retrospective data collected in 2 separate studies. However, both studies were conducted by the same research group and had the same study design. It was already decided in the development phase that the data would be merged for an overarching study; however, we could not include more questions about factors related to program use in the questionnaires. The interviews were conducted to retrieve patients’ experiences, not to identify program characteristics that influence program use. Vascular View and Coping with RA were developed based on BCTs, and most of these were unobtrusively included in the program. In addition, Vascular View and Coping with RA applied different implementation strategies that could have influenced program use, making the programs harder to compare. Therefore, the program characteristics identified in this study are *potential* mechanisms and should be tested in future research. Another limitation was that *physical comorbidity* and *time since diagnosis* were measured using a questionnaire. Although this provides insight into patients’ experiences, the self-reporting of clinical variables is not always reliable. The last limitation of this study was that we only included participants who had access to the internet and an email address. This may have biased our results by excluding people with a very low level of eHealth literacy. However, the internet is easily accessible in the Netherlands (97% of households have access to the internet [44]), so most people would have been able to participate.

### Conclusions

This realistic evaluation identified contexts and potential mechanisms, in the form of patient, disease, and program characteristics, that are associated with the use of web-based self-management support programs. Our results emphasized the importance of (1) tailoring interventions to patients’ needs (depending on age, education, and program characteristics) to increase program use and (2) considering whether all patients can use eHealth interventions (depending on disease burden and eHealth literacy) and providing alternative self-management support when needed. These results are a first step toward improving the tailoring and use of web-based self-management support programs. Future research into the interaction between patient and program characteristics and how this affects program use should be conducted to improve the tailoring of participants to program components.

## Appendix

Multimedia Appendix 1 Description of the web-based self-management support programs Vascular View and Coping with Rheumatoid Arthritis.

## Abbreviations

BCT: behavior change technique
CVD: cardiovascular disease
PAM-13: Patient Activation Measure
RA: rheumatoid arthritis
RAND-36: RAND 36-item health survey
SeMaS: Self-Management Screening
