# Supplementary material for: The Influencing Contexts and Potential Mechanisms Behind the Use of Web-Based Self-management Support Interventions: Realistic Evaluation
Source: JMIR Hum Factors. 2022 Jul 1;9(3):e34925. doi: 10.2196/34925 (PMC9288100; doi:10.2196/34925)
Supplement: Multimedia Appendix 1 [file humanfactors_v9i3e34925_app1.docx]

**Multimedia appendix 1: Description of the online self-management support programs Vascular View and Coping with Rheumatoid Arthritis**

|  | **Vascular View** | **Coping with Rheumatoid Arthritis** |
| --- | --- | --- |
| Theory | Integrated change model 2.0 | Theory of planned behavior |
| Modules | 6 modules (3-4 sessions each): - Coping with cardiovascular disease - Setting boundaries in daily life  - Lifestyle  - Healthy nutrition  - Being physically active  - Interaction with health professional | 9 modules (2-5 sessions each): - Balancing activity and rest - Setting boundaries - Asking for help and social support - Using medicines - Communicating with health professionals - Using assistive devices - Performing physical exercises - Coping with worries - Coping with RA |
| Tailoring | Assessment at start of the program to receive tailored advice about which module(s) were recommended | The content of the modules is tailored to the user’s self-efficacy |
| Practical applications | Written information, tailored feedback, quotes from and videos of patients, pictures, exercises | Informational and persuasive texts, videos with instructions and role models, exercises, assignments |
| Diaries | Two diaries (exercise and nutrition) to register behavior and get insight into routines | A diary to track fatigue and pain over time |
| Implementation | 1) Patients receive a written instruction manual 2) Email reminder after first month if patient hasn’t logged in. Phone call reminder one week later  3) Email reminder after visiting program but not finishing a module 4) A digital newsletter every 2 months with information about the program and contact details | 1) Patients received a written instruction manual 2) Email reminders to (re)visit the program twice weekly  3) Nurses tell patients about the program during consultations |
